# Endothelial Atg7 Deficiency Ameliorates Acute Cerebral Injury Induced by Ischemia/Reperfusion

**DOI:** 10.3389/fneur.2018.00998

**Published:** 2018-11-28

**Authors:** Hui-Jie Wang, Jia-Yi Wei, Dong-Xin Liu, Shi-Fang Zhuang, Yuan Li, Hui Liu, Meng Ban, Wen-Gang Fang, Liu Cao, Wei-Dong Zhao, Yu-Hua Chen

**Affiliations:** Key Laboratory of Cell Biology, Ministry of Public Health, and Key Laboratory of Medical Cell Biology, Ministry of Education, Department of Developmental Cell Biology, China Medical University, Shenyang, China

**Keywords:** Atg7, brain microvascular endothelial cells, ischemia/reperfusion injury, pro-inflammatory cytokines, transient focal cerebral ischemia

## Abstract

Ischemic strokes often result in cerebral injury due to ischemia/reperfusion (I/R). Although the local inflammatory responses are known to play a primary role in the brain I/R injury, the underlying mechanism remains unclear. In the current study, we investigated the effect of brain endothelial Atg7 (autophagy related 7) depletion in the acute brain injury induced by ischemia and reperfusion. Endothelial knockout of Atg7 in mice (Atg7 eKO) was found to significantly attenuate both the infarct volume and the neurological defects induced by I/R when compared to the controls. In fact, brain inflammatory responses induced by I/R were alleviated by the Atg7 eKO. Furthermore, an increased expression of pro-inflammatory cytokines, including IL-1β, IL-6, IL-8, and TNF-α, was observed in brain endothelial cells in response to oxygen/glucose depletion/reoxygenation, which was decreased by the shRNA-mediated Atg7 knockdown. Interestingly, Atg7 knockdown reduced IKKβ phosphorylation, leading to NF-κB deactivation and downregulation of the pro-inflammatory cytokines mRNA levels. Further, Atg7 transcriptional regulation function is independent of its role in autophagy. Taken together, our results demonstrated that brain endothelial Atg7 contributes to brain damage during I/R by modulating the expression of pro-inflammatory cytokines. Depletion of Atg7 in brain endothelium has a neuroprotective effect against the ischemia/reperfusion-induced acute cerebral injury during stroke.

## Introduction

Stroke is one of the leading causes of mortality and disability worldwide (1). Specifically, the ischemic stroke accounts for approximately 87% of all strokes (2) and occurs when the cerebral blood flow (CBF) is locally interrupted or blocked within a vessel (3). As the occluded vessel is recanalized, the reestablishment of circulation could initiate cerebral reperfusion in both stroke patients and experimental stroke models (3, 4). The rapid restoration of blood flow and reoxygenation is frequently associated with an exacerbation of tissue injury, called ischemia/reperfusion (I/R) injury (5). Multiple mechanisms that account for the brain I/R injury were identified, including excitotoxicity (6), free radical release (7), inflammatory responses (8), shortage of energy supply (9), and ischemic apoptosis or necrosis (10).

Furthermore, increasing evidence suggested a primary role for inflammatory responses in the brain I/R injury (11). In fact, the brain responds to I/R injury with an inflammatory process, characterized by rapid activation of resident cells (mainly microglia) (12, 13), infiltration of neutrophil into the ischemic brain parenchyma (14, 15) and production of pro-inflammatory cytokines, such as interleukin (IL)−1β, IL-6, IL-8, TNF (tumor necrosis factor) -α, chemokine (C-C motif) ligand 2 (CCL2), chemokine (C-X-C motif) ligand 1 (CXCL1) (8, 11, 16). Additionally, inhibiting the expression of pro-inflammatory cytokines after stroke was shown to both reduce brain damage and improve the neurological outcome of I/R injury in rodent stroke models (17–20). These evidences indicated that an increase in pro-inflammatory cytokines production is necessary for the pathogenesis of ischemic stroke and reperfusion injury.

Cerebral blood vessels are one of the first major tissues to be affected during the rapid reperfusion of I/R (1). However, the role of cerebral endothelium in I/R injury is yet poorly understood. Recently, an increase in CXCL-10 expression in the vascular endothelia was observed in the spinal cord of the experimental allergic encephalomyelitis (EAE) model, suggesting the involvement of brain endothelium in cerebral inflammatory responses (21). Further, *in vitro* studies reported that the expression of IL-1β in mouse brain microvascular endothelial cells (mBMECs) was increased in the oxygen-glucose deprivation/reoxygenation (OGD/R) condition, mimicking the *in vivo* I/R injury (22). Increased mRNA expression of TNF-α, IL-1β, and IL-6 was reported in mBMECs with 4 h of OGD (23), raising the possibility that the brain endothelium may be associated with the release of both pro-inflammatory cytokines and the related inflammatory responses during I/R injury.

Atg7 (autophagy related 7) is an ubiquitin-activating E1-like enzyme and is crucial for both the autophagy conjugation complex and the autophagosome formation (24). Additionally, Atg7 can activate Atg12, which is essential for Atg12 and Atg5 conjugation. Further, LC3-I (microtubule-associated protein 1 light chain 3) may be activated by Atg7, which is vital for the formation of phosphatidylethanolamine-conjugated LC3 and autophagosome (25). Besides its autophagic functions, Atg7 plays an important role in non-autophagic processes, including adipogenesis (26), pulmonary fibrosis (27), and *Klebsiella pneumonia* infection (28). The role of Atg7 in ischemic stroke and reperfusion injury has been controversial in previous studies (29, 30, 31). In fact, although Atg7 deficiency in neurons was reported to provide neuroprotection from brain injury in mice (29), Atg7 silencing aggravated ischemia-induced brain damage (30, 31). Therefore, the role of brain endothelial Atg7 in I/R injury is yet to be identified.

In the current study, endothelium-specific Atg7 knockout (Atg7 eKO) mice were used to assess the role of endothelial Atg7 in brain injury caused by ischemia/reperfusion. As the interesting protective effect of Atg7 deficiency against both brain damage and release of pro-inflammatory cytokines was revealed, further investigations on the mechanism behind Atg7 regulation of pro-inflammatory cytokines expression in brain endothelial cells were prompted.

## Materials and methods

### Animals handling

The construction of endothelial Atg7 knockout mice (Atg7 eKO) on a C57/Bl6 genetic background and the identification of the genotyping procedure were described previously (32–34). In brief, the mice harboring LoxP-flanked *Atg7* were crossed with transgenic mice expressing Cre recombinase under the control of the vascular endothelial cadherin (VE-cadherin) promoter to generate mice with endothelial-specific Atg7 knockout. The mice were raised in a 12-h dark-light cycle condition, with food and water available *ad libitum*. All the animal experimental procedures were conducted in accordance with the regulations of the animal protection laws of China and were approved by the Animal Experimentation Ethics Committee of the China Medical University (protocol#: 14031).

### Transient focal cerebral ischemia and reperfusion (tFCI/R) model and experimental groups

Adult male mice (6~8 weeks of age, 20 ± 1 g) were anesthetized with an intraperitoneal injection of 0.8% pentobarbital sodium (40 mg/kg). Following an incision in the neck midline, the right carotid triangle, common carotid artery (CCA), carotid bifurcation, external carotid artery (ECA), and internal carotid artery (ICA) were isolated through blunt dissection. A 6-0 monofilament (Doccol Corporation, Sharon, MA, USA) with a silicon-coated tip was introduced into the ICA via a cut in the ECA, distal to the bifurcation, until the monofilament slowly advanced and occluded the origin of the middle cerebral artery (MCA). Successively to a 60-min occlusion, the monofilament was gently withdrawn to permit reperfusion. Mice body temperature was maintained at 37.0 ± 0.5°C during the surgery with a heating pad. Furthermore, the regional CBF was measured using the Laser Doppler flowmetry (Perimed, Jarfalla, Sweden). Additionally, animals not showing a CBF reduction of (at least) 75% from the baseline level after the MCA occlusion, or presenting subarachnoid hemorrhages, or that died following reperfusion were excluded from further experiments. With regard to the sham-operated animals, anesthesia and all surgical procedures were identical to the ones conducted on the experimental animals, with the exception of the MCA occlusion. Animals were randomized into four experimental groups: (1) WT littermate mice for sham operation (WT, sham), (2) WT littermate mice for tFCI (WT, tFCI/R24h), (3) Atg7 eKO mice for sham operation (Atg7 eKO, sham), and (4) Atg7 eKO mice for tFCI (Atg7 eKO, tFCI/R24h).

### Neurobehavioral tests

Neurological deficits were evaluated with a modified protocol according to Longa's method of the five-point scale assessment (35–37), i.e., 0 point: no neurological deficit, mice behave normally; 1 point: mice cannot fully stretch their left front legs; 2 points: mice turn around into a circle; 3 points: mice fall down to the left side; 4 points: mice cannot move by themselves, losing their consciousness.

The rotarod test was performed to assess post-stroke motor functions (20, 38). Specifically, mice were placed on a rotating drum and the time at which the mouse fell off the drum (latency to fall) was recorded. The test began 3 days prior to the surgery, while five trials were performed on each mouse on the day of the surgery and the mean value of trials 3–5 was used as the pre-surgery baseline value. Subsequently, mice were tested for additional five trials 24 h after the surgery, with 15-min intervals, and the data for trials 3–5 were used to calculate the mean latency prior to the fall.

The pole test was performed as previously described (20, 39), with minor modifications. A vertical wood pole (diameter of 1 cm, height of 50 cm) was placed inside the cage and was covered with tape to create a rough surface. The total time spent by the mouse to transport from the top of the pole to the bottom of the cage with the front paws was recorded. Briefly, the test consisted of five trials on the testing day, with 5-min intervals, and the data were expressed as the mean value of trials 3–5. Moreover, pre-surgery training was performed 3 days prior to the MCAO surgery and the mean value of trials 3–5 was used as the pre-surgery baseline value. Thereafter, mice were tested 24 h following the surgery. Surgeries and all stroke outcome assessments were performed by investigators blinded to both the mouse genotype and the experimental group allocation.

### TTC staining and evaluation of infarct volume

All mice were sacrificed for the assessment of their brain infarct volume at 24 h following the onset of tFCI. Brains were quickly removed, placed in a brain matrix, and sliced into 2-mm coronal sections. Subsequently, they were stained in a 2% 2,3,5-triphenyltetrazolium chloride (TTC, Sigma-Aldrich, St. Louis, MO, USA) solution for 60 min and were then fixed with 4% paraformaldehyde overnight. The normal viable brain tissue was stained red, whereas the infarct region remained white. Further, the infarct volume was analyzed using 4 slices of 2-mm coronal sections from each brain and calculated with the following formula: Percentage of corrected infarct volume = [Contralateral hemisphere area – (Ipsilateral hemisphere area – Measured infracted area)]/Contralateral hemisphere area × 100% (40).

### Cresyl violet staining

The slices were fixed again in a 4% paraformaldehyde solution for 10 min and then briefly rinsed in double-distilled water. Successively, the slices were stained in a cresyl violet solution for 15 min and washed in double-distilled water twice. Additionally, they were immersed twice in 95% ethanol for 2 min and twice in fresh xylene for 5 min. Finally, the slices were cover-slipped for observation and long-term storage.

### Terminal deoxynucleotidyl transferase-mediated dutp nick end labeling (TUNEL) analysis

TUNEL analysis was conducted according to the manufacturer's instructions (Roche, Mannheim, Germany). The brain coronal slices were incubated in the TUNEL reaction mixture for 1 h at 37°C. Thereafter, the nuclei were counterstained with 4,6-diamidino-2-phenyl-indole (DAPI, 0.5 μg/mL). Finally, fluorescent images were captured with the Olympus BX51 fluorescence microscope (Olympus, Tokyo, Japan).

### Immunofluorescence analysis

The brain slices were incubated with PBS containing 0.3% Triton X-100 and 5% bovine serum albumin (BSA) for 1 h at room temperature. Subsequently, they were incubated with the corresponding primary antibodies against either Atg7 (1:100, Abcam) or Ly6B.2 (1:50, Abcam), and the CD31 antibody (1:200, R&D system) overnight at 4°C, respectively. The slices were then incubated with the secondary antibodies (1:200, Invitrogen, Carlsbad, CA, USA) for 1 h at room temperature. Thereafter, slices were counterstained with DAPI for 5 min and sealed with the antifade Vectashield solution (Vector Laboratories, Burlingame, CA, USA). Finally, fluorescent images were captured with the confocal laser scanning microscope (Zeiss 880, Oberkochen, Germany). In contrast, for immunostaining of either NF-κB or HIF-1 in HBMECs, cells were fixed and incubated with the antibody against NF-κB p65 (1:100, CST) and HIF-1α (1:100, Novus), respectively.

### Cell culture

Human brain microvascular endothelial cells (HBMECs) (41) were cultured in the RPMI 1640 medium, supplemented with 10% fetal bovine serum (Invitrogen, Logan, UT, USA), 10% Nu-serum (BD Biosciences, San Jose, CA, USA), 2 mM glutamine, 1 mM sodium pyruvate, 1 X nonessential amino acids, and 1 X MEM vitamin. The cells were incubated at 37°C in 5% CO_2_ and humidified 95% air.

### RNA interference

The small hairpin RNA (shRNA) sequence targeting the coding region of human Atg7 (NM_006395) used was GGTCAAAGGACGAAGATAA (781–799), whereas a non-silencing shRNA sequence (TTCTCCGAACGTGTCACGT) (24898658) was employed as the control. For the establishment of stable HBMECs with Atg7 knockdown, vectors containing the Atg7 shRNA were transfected into HBMECs, as previously described, with non-silencing shRNA as the control (42). Furthermore, stable cell clones were selected with G418 (300 μg/mL, Invitrogen).

When appropriate, HBMECs were transiently transfected with small interfering RNA (siRNA) targeting human Atg7 using Lipofectamine 2000 (Invitrogen), according to the manufacturer's instructions, with non-silencing siRNA as the control. At 48 h following this procedure, the transfected HBMECs were employed in subsequent experiments. The knockdown effect was assessed by western blot analysis with the Atg7 antibody. The targeting sequence of Atg7 siRNA and non-silencing siRNA were identical to that of Atg7 shRNA and non-silencing shRNA, respectively.

### Oxygen-glucose deprivation followed by reoxygenation (OGD/R)

The HBMECs was cultured in glucose-free RPMI 1640 medium in an oxygen-deprived incubator (1% O_2_, 5% CO_2_, 94% N_2_) at 37°C for 4 h. Following, cells were returned to the normoxic condition with the regular culture medium to terminate OGD and start reoxygenation at 37°C for 24 h.

### Cell viability assay

Cultured HBMECs were assessed for metabolic viability using the CCK-8 assay kit (Beyotime, Shanghai, China). Cells in 96 well-plates were incubated with the CCK-8 reaction mixture at 37°C for 1 h. Successively, the medium was carefully removed to a new 96 well-plate for measurement. Absorbance at 450 nm was measured with a microplate reader (Molecular Devices, Sunnyvale, California, USA).

### Enzyme-linked immunosorbent assay (ELISA)

Mice were sacrificed and their brain cortex and ipsilateral and contralateral hemisphere striatum were homogenized using the RIPA lysis buffer (Beyotime), according to manufacturer's instructions. The counterparts of the contralateral hemisphere were employed as the negative control. The protein levels of IL-1β, IL-6, IL-8, and TNF-α were determined by the ELISA kits (Invitrogen), according to the manufacturer's instructions.

Furthermore, the secretion of IL-6, IL-8, and TNF-α in the culture supernatants of HBMECs, as well as, the expression of IL-1β in the cell lysates of HBMECs were determined by the ELISA kits (R&D System, Minneapolis, MN, USA) according to the manufacturer's instructions. Absorbance at 450 nm was read with a microplate reader (Molecular Devices).

### Western blot analysis

Total proteins were extracted with the RIPA lysis buffer and their concentration was determined using a BCA kit (Pierce, Rockford, IL, USA). A 30-μg aliquot of proteins from each sample was separated with the SDS-PAGE and subsequently transferred to a PVDF membrane (Millipore, Billerica, MA, USA). The membranes were blocked with 5% non-fat milk at room temperature for 1 h and incubated with primary antibodies against Atg7 (1:1,000, Sigma-Aldrich), phosphor-IKKβ (1:1,000, CST), IKKβ (1:500, Abcam), phosphor-IκBα (1:1,000, CST), IκBα (1:1,000, CST), HIF-1α (1:500, Novus), NF-κB p65 (1:1,000, CST), β-tubulin (1:5,000, Abmart), β-actin (1:5,000, ZSGB-BIO), or Fibrillarin (1:2,000, Abcam), at 4°C overnight, with gentle shaking. Where appropriate, either the β-tubulin, β-actin, or Fibrillarin were used as the loading controls. All the membranes were washed and incubated with the horseradish peroxidase (HRP)-conjugated secondary antibody (1:5,000, EarthOx, San Francisco, CA, USA) for 1 h. Thereafter, immunoreactive bands were visualized by the SuperSignal West Pico Chemiluminescent Substrate (Pierce) and imaged with the LAS-3000mini Imaging system (Fujifilm, Tokyo, Japan). Finally, signal densities were analyzed with the ImageJ software (National Institutes of Health, Bethesda, MD, USA).

### Real-time PCR

Mice were sacrificed and their brain cortex and striatum were isolated and homogenized using the TRI Reagent (Sigma-Aldrich). For HBMECs, the total RNA was extracted from the cells with the TRI Reagent. The first-strand cDNA was generated from 2 μg of the isolated total RNA using the FastQuant cDNA synthesis kit (Tiangen, Beijing, China), according to the manufacturer's instructions. Appropriate primers were designed and are listed in Supplementary Table 1. All reactions were performed on an ABI 7500 real-time PCR system (Thermo Fisher Scientific, Waltham, MA, USA) with the SuperReal Premix Plus kit (Tiangen), according to the manufacturer's instructions. Finally, real-time PCR products were analyzed with agarose gel electrophoresis, while their relative expression levels were estimated using the ΔΔCT method, with β-actin representing the internal control to normalize gene expression.

### Chromatin immunoprecipitation (CHiP)

Chromatin immunoprecipitation was performed according to the protocol of the EZ-ChIP kit (Millipore). Cells (1 × 10^7^) were cross-linked with 1% formaldehyde, while the chromatin was sonicated into fragments. Following, the fragmented chromatin was incubated and immunoprecipitated using the anti-p65 antibody, with the IgG acting as the control. Successively to the decrosslink, the immunoprecipitated DNA was eluted and subjected to real-time PCR analysis. All quantitative PCR primers for the ChIP are shown in Supplementary Table 2.

### Statistical analysis

Data are expressed as the means ± SD of three independent experiments. Statistical significance between two groups was analyzed through the unpaired two-tailed Student's *t*-test. In contrast, one-way analysis of variance (ANOVA), followed by the Dunnett's test, was performed for multiple groups comparison. Differences were considered statistically significant at *P* < 0.05.

## Results

### Endothelial Atg7 deficiency ameliorates ischemia/reperfusion-induced brain damage

**Figure 1:**
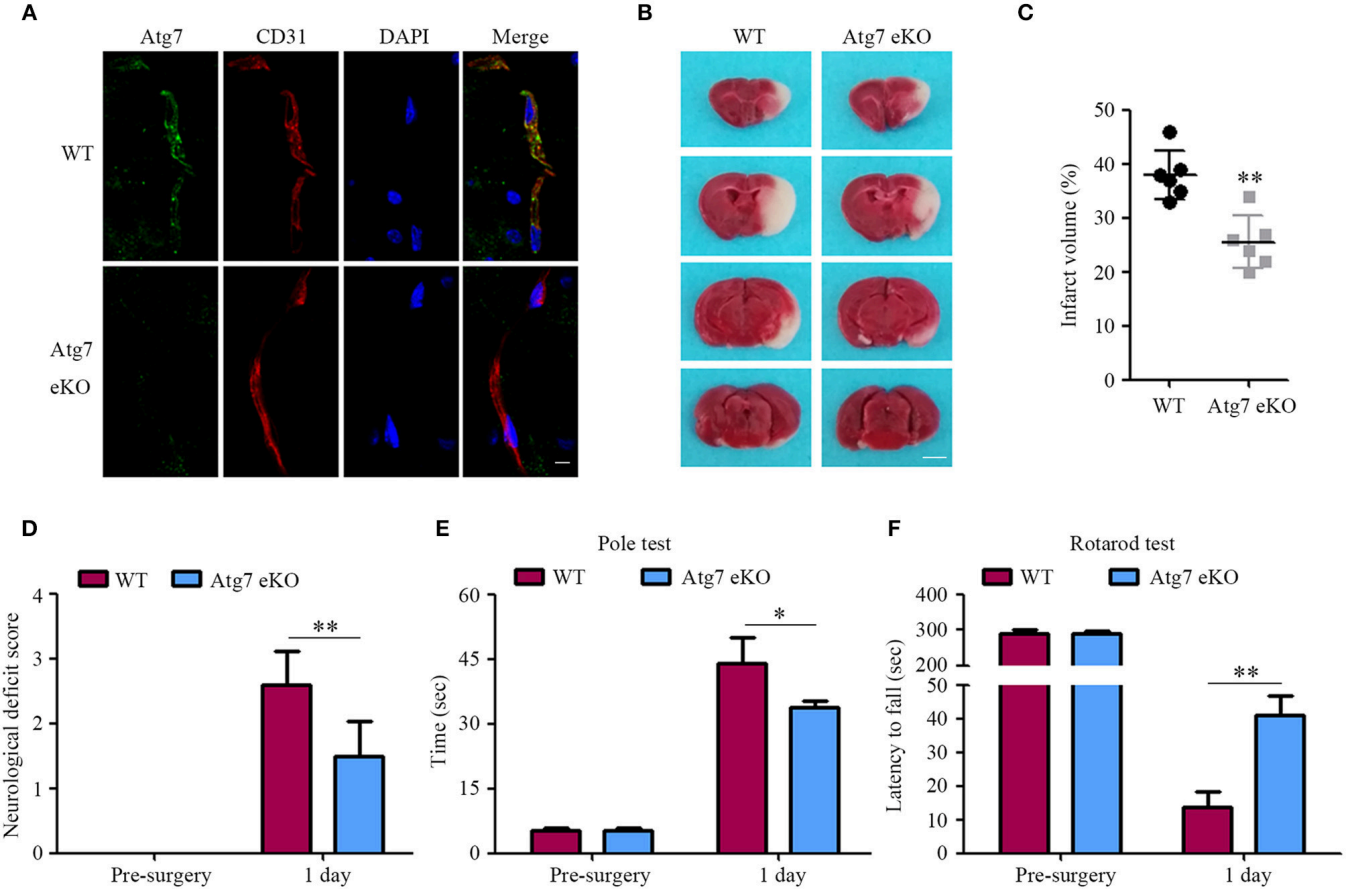
Endothelial-specific Atg7 deletion ameliorates the infarct volume and the neurological deficits in mice with cerebral I/R injury. **(A)** The tissue samples were obtained from transgenic mice with endothelial-specific Atg7 knockout (Atg7 eKO), and from littermate wild-type (WT) mice as the controls. Following the preparation of the brain slices, immunofluorescence was performed with the primary antibody against Atg7 (green) and CD31 (red). DAPI was used for counterstaining. The stained slices were examined under confocal microscope (*n* = 10 brain slices from 3 different mice). The representative images from three independent experiments were presented. Scale bar, 10 μm. **(B)** The mice were subjected to tFCI followed by a 24-h reperfusion. Then the mice were sacrificed and their coronal brain sections were stained with 2% TTC. The representative images are presented, in which the white parts indicate the infarcted area (*n* = 6 mice per group). Scale bar, 2 mm. **(C)** To quantify the results in **(B)**, brain infarct volumes were measured with the ImageJ software and the percentage of infarct volume to the contralateral hemisphere was calculated. ^**^P < 0.01. **(D)** For quantification of the neurological deficits, mice were assessed by the five-point scale and the deficit score was calculated. ^**^P < 0.01. **(E,F)** Mice sensorimotor deficits were evaluated by the pole test **(E)** and the rotarod test **(F)** at day 3 prior to and day 1 following the 1-h tFCI, respectively. ^*^P < 0.05, ^**^P < 0.01 (*n* = 6 mice per group).

Atg7 deficiency in the cerebral blood vessels of Atg7 eKO mice was verified by immunofluorescence of brain slices. Specifically, brain endothelial cells were stained with CD31, also known as platelet endothelial cell adhesion molecule (PECAM-1), which is a specific marker for endothelial cells. When compared to WT littermate mice, Atg7 was absent in Atg7 eKO mice brain endothelium (Figure 1A), indicating successful genetic deletion of Atg7 in cerebral blood vessels. Successively, mice were subjected to transient focal cerebral ischemia (tFCI) for 1 h followed by reperfusion for 24 h. The brain infarct volume was evaluated by TTC staining; specifically, the TTC negative (white) area in the ipsilateral cerebral regions was calculated over a series of brain sections. Interestingly, we found the infarct volume in the Atg7 eKO mice brain sections to be significantly reduced compared to that in WT littermate mice (*P* < 0.01, *n* = 6, Figures 1B,C). Subsequently, mice were examined and scored for neurological deficits using a five-point scale, as described previously (35–37). WT mice showed marked neurological behavioral deficits, while attenuated neurological deficits were observed in Atg7 eKO mice (Figure 1D). To further evaluate the motor function, both the pole and rotarod tests were performed 24 h after the reperfusion following the tFCI, while baseline measurements were performed prior to the tFCI. The results from the pole test reported that Atg7 eKO mice took a shorter time than WT control mice to move along the pole, from the top to the bottom (Figure 1E). Similarly, Atg7 eKO mice presented significant longer latency on the rotating rod than WT control mice (Figure 1F). These results indicated that Atg7 knockout in cerebral vessels attenuated the brain damage induced by cerebral ischemia/reperfusion.

### Neuronal death induced by ischemia/reperfusion is attenuated by Atg7 knockout in the endothelium

**Figure 2:**
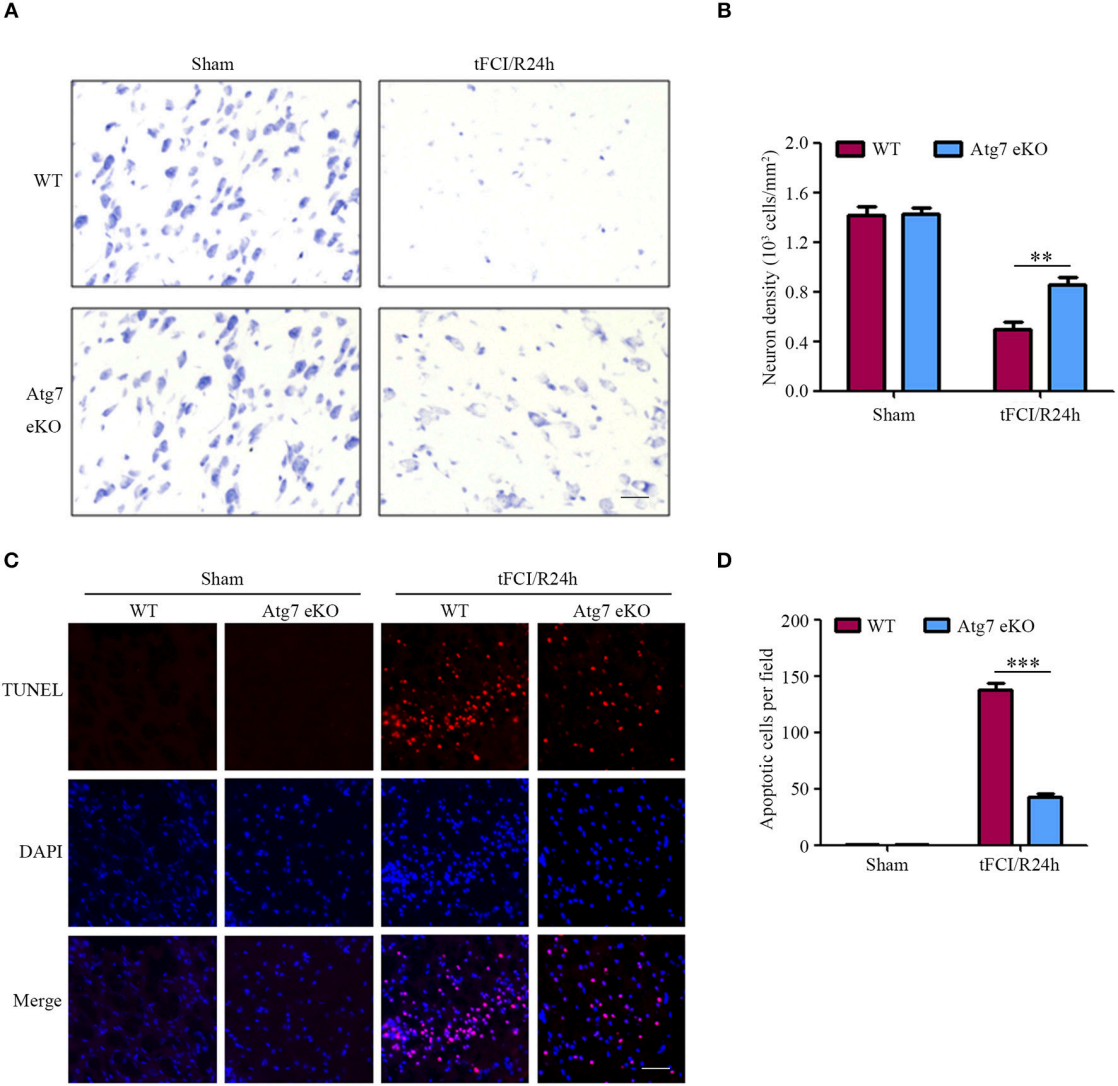
Endothelial-specific Atg7 knockout alleviates neuronal death and brain cell apoptosis induced by I/R. WT and Atg7 eKO mice were subjected to either a sham operation or a 1-h tFCI followed by a 24-h reperfusion (tFCI/R24h). **(A)** Cresyl violet staining was performed on the brain slices and the images were captured. The representative images from the striatal regions from three independent experiments were shown. Scale bar, 20 μm. **(B)** To quantify the results in **(A)**, the numbers of necrotic neurons were counted by ImageJ software and neuron density was calculated. ^**^P < 0.01. **(C)** TUNEL assays were performed with the prepared brain slices and the images were captured by fluorescence microscopy. The representative images from three independent experiments were shown. Scale bar, 100 μm. **(D)** To quantify the results of the TUNEL assays in **(C)**, the number of apoptotic cells were counted by ImageJ software. ^***^P < 0.001.

To further determine the brain neuronal damage, cresyl violet staining was performed to assess the neuronal morphology and cytoarchitecture in brain slices harvested 24 h after reperfusion. In sham-operated mice, differences in the density and morphology between neurons in the striatum were not observed (Figures 2A,B). In contrast, neurons of those mice challenged with tFCI followed by 24-h reperfusion appeared smaller and pyknotic, with a reduced density in the penumbra of the cerebral striatum (Figures 2A,B). Interestingly, both the morphological changes and the reduced density of neurons, found in WT mice in response to tFCI, were significantly alleviated in Atg7 eKO mice (Figures 2A,B). These results suggested that endothelial Atg7 knockout has a protective effect on neuronal cell death induced by I/R injury. Successively, we performed TUNEL staining to further identify the brain apoptotic cells. Cell apoptosis was hardly detectable in the sham-operated groups, whereas apparent apoptotic cell death was observed in the penumbra of the brain cortex in response to I/R injury (Figures 2C,D) in the experimental groups. In line with the results reported in Figures 2A,B, we found that Atg7 eKO significantly reduced the number of apoptotic cells in the penumbra compared to the WT littermate mice (Figures 2C,D). These results indicated that endothelial Atg7 knockout protected against neuronal apoptosis induced by a 24-h reperfusion injury.

### Endothelial Atg7 deficiency attenuates the inflammatory responses in the post-ischemic brain

Previous studies revealed that cerebral vascular angiogenesis contributed to the repairing process which follows I/R injury in rodent models (43, 44). However, the cerebral vasculature in the penumbra of the brain striatum did not present any significant difference between the WT littermate and Atg7 eKO mice in response to I/R in our study (Supplementary Figures 1A,B), suggesting that cerebral vascular angiogenesis is not responsible for the protecting effect of endothelial Atg7 knockout.

**Figure 3:**
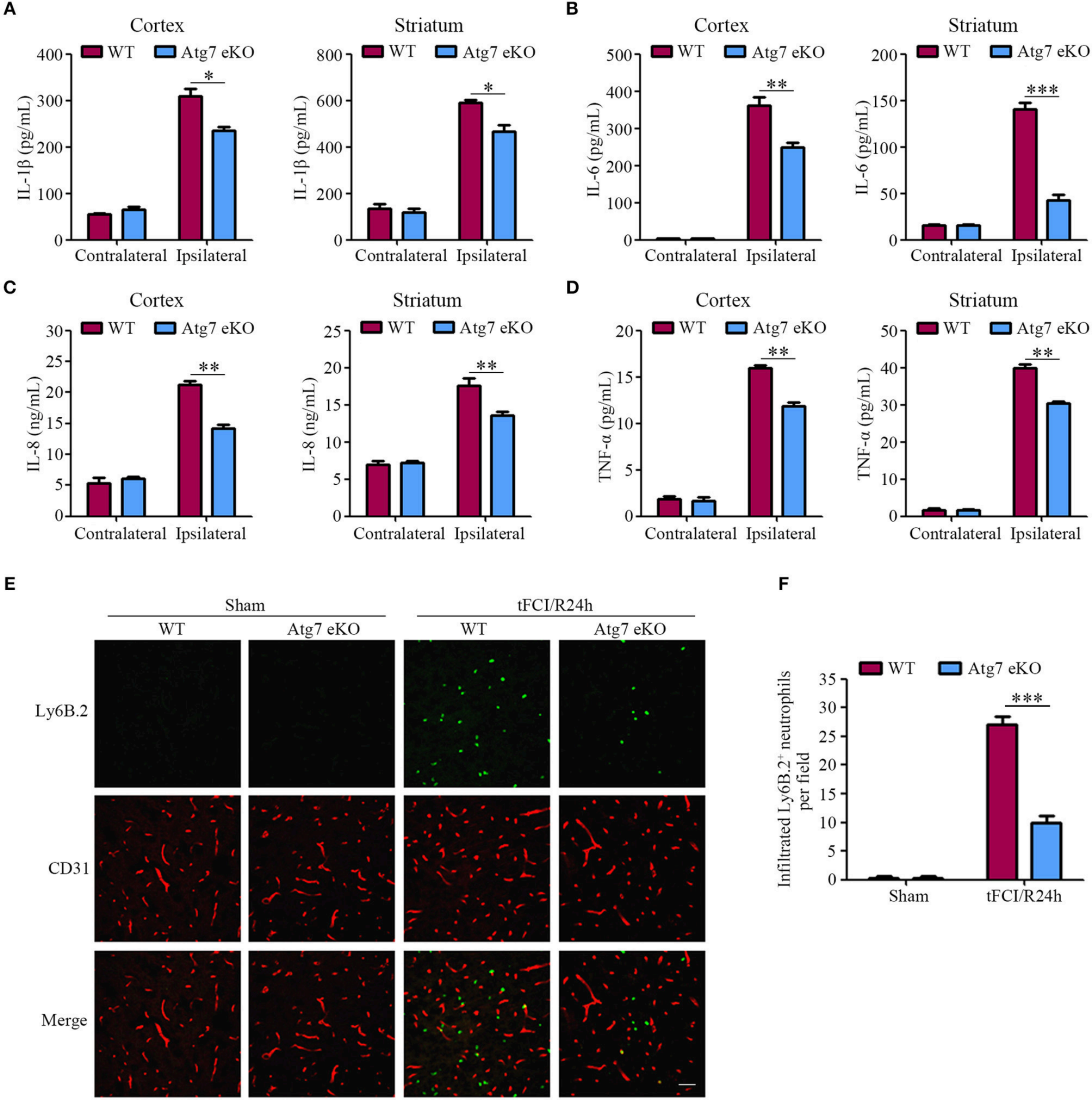
Endothelial Atg7 deletion suppresses the upregulation of pro-inflammatory cytokines and impedes neutrophil infiltration in the brain in response to I/R. **(A–D)** The mice were subjected to either a sham operation or a 1-h tFCI followed by a 24-h reperfusion. The protein levels of the pro-inflammatory cytokines, including IL-1β, IL-6, IL-8, and TNF-α, in the contralateral and ipsilateral cerebral cortex, as well as, the striatum homogenates were measured by ELISA (*n* = 6 mice per group). ^*^P < 0.05, ^**^P < 0.01, ^***^P < 0.001. **(E)** Immunofluorescence was performed with the primary antibody Ly6B.2 (neutrophil marker, green) and CD31 (red) on the brain slices. DAPI was counterstained for nucleus labeling. The stained slices were examined under a confocal microscope (*n* = 10 brain slices from 3 different mice). Scale bar, 100 μm. **(F)** To quantify the results in **(E)**, the number of infiltrated Ly6B.2^+^ neutrophils in the brain parenchyma were counted by ImageJ software. ^***^P < 0.001.

Furthermore, given that inflammatory responses were considered to play a significant role in stroke pathology (45–47), the expression of pro-inflammatory cytokines, including IL-1β, IL-6, IL-8, and TNF-α, was measured in the contralateral and ipsilateral cortex, as well as, the striatum homogenates at 24 h post-tFCI. The results reported the protein levels of IL-1β, IL-6, IL-8, and TNF-α in the ipsilateral hemispheres homogenates to be significantly increased in response to tFCI, when compared to the contralateral counterparts (Figures 3A–D). Interestingly, the levels of IL-1β, IL-6, IL-8, and TNF-α in the ipsilateral cortex and striatum homogenates were found to be reduced in Atg7 eKO mice when compared to those in WT mice (Figures 3A–D). Similarly, the mRNA levels of IL-1β, IL-6, and TNF-α were reduced in Atg7 eKO mice which suffered from I/R when compared to WT mice, whereas the IL-8 mRNA was slightly affected (Supplementary Figures 2A–D). These results suggested that endothelial Atg7 deficiency attenuated the increased expression of pro-inflammatory cytokines in the brain induced by a 24-h reperfusion.

Additionally, the infiltration of neutrophils into the brain parenchyma is crucial in the pathological process of the inflammatory response induced by I/R (48). Our results showed that the number of neutrophils in the ipsilateral hemispheres was dramatically increased when compared to the sham-operated mice, reflecting the robust infiltration of blood-derived neutrophils into the brain parenchyma in response to tFCI (Figures 3E,F). In contrast to WT littermate mice, the tFCI-induced infiltration of neutrophils was significantly reduced in Atg7 eKO mice (Figures 3E,F). These results indicated that the inflammatory responses induced by the 24-h reperfusion were ameliorated by endothelial Atg7 knockout.

### Silencing Atg7 in brain endothelial cells reduced the expression of pro-inflammatory cytokines

**Figure 4:**
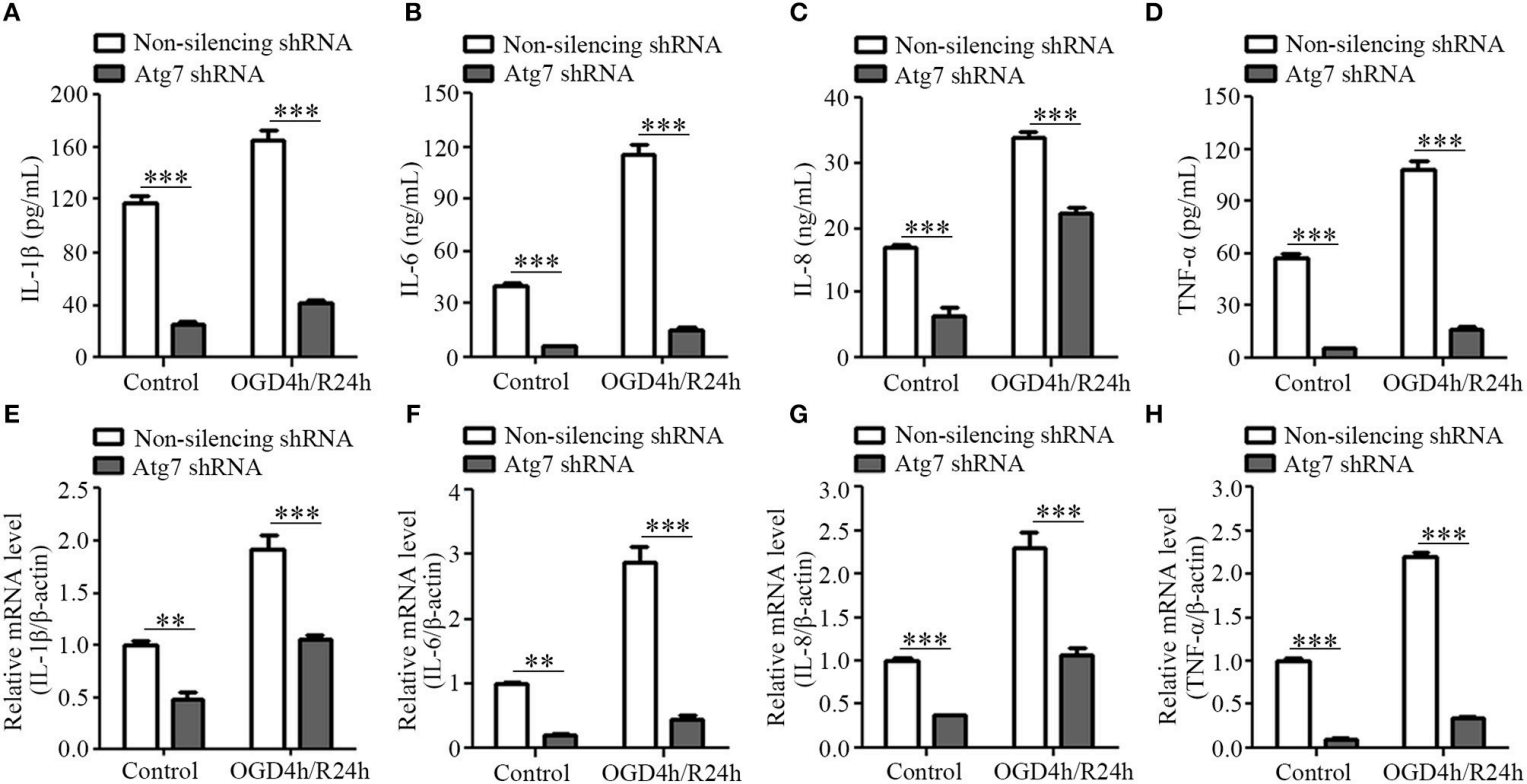
Atg7 knockdown inhibits the expression of pro-inflammatory cytokines in the brain endothelial cells. HBMECs were subjected to oxygen-glucose depletion for 4 h followed by reoxygenation for 24 h (OGD4h/R24h), with cells cultured under normoxia condition as control. The HBMECs stably transfected with Atg7-specific shRNA were compared with those HBMECs transfected with non-silencing shRNA. **(A)** IL-1β expression in the cell lysates of HBMECs was determined by ELISA. ^***^P < 0.001. **(B–D)** The concentrations of IL-6, IL-8, and TNF-α in the supernatant of HBMECs were determined by ELISA. **(E–H)** The mRNA levels of the pro-inflammatory cytokines in the HBMECs were determined by real-time PCR. ^**^P < 0.01, ^***^P < 0.001.

To further determine the molecular relationship between Atg7 and the release of pro-inflammatory cytokines, stable Atg7-silenced cell lines were established with HBMECs transfected with shRNA targeting Atg7, while non-silencing shRNA served as the control. The knockdown effect was analyzed by western blot and Atg7 protein levels were found to be significantly decreased when compared to the non-silencing shRNA control (Supplementary Figure 3A). Cell viability was first assessed with CCK-8 reagent, which reported an absence of significant difference between the Atg7-silenced cells and non-silencing control cells within the 4-h oxygen-glucose deprivation (OGD) (Supplementary Figure 3B). Subsequently, the 4-h OGD treatment was chosen to mimic the *in vivo* ischemia-like insult. The cells were further subjected to reoxygenation to establish the OGD/R model, which simulated the *in vivo* ischemia/reperfusion conditions. Our results reported a significantly lower IL-1β expression in the cell lysates of the stable HBMEC cell lines with silenced Atg7 than in the non-silencing shRNA controls (Figure 4A). Similarly, the concentration of IL-6, IL-8, and TNF-α in the media of the Atg7-silenced HBMECs was significantly lower than the controls (Figures 4B–D). Furthermore, both IL-1β expression in the cell lysates and the concentration of IL-6, IL-8, and TNF-α in the media were dramatically increased in response to the 4-h OGD followed by 24-h reoxygenation (OGD4h/R24h); however, this was significantly reduced by Atg7 knockdown in HBMECs (Figures 4A–D). In concordance, the mRNA levels of IL-1β, IL-6, IL-8, and TNF-α in HBMECs were reduced with Atg7 knockdown (Figures 4E–H). Similar results were obtained with HBMECs transiently transfected with Atg7-specific siRNA (Supplementary Figures 4A–E). Overall, these data indicated that Atg7 knockdown inhibits the expression of pro-inflammatory cytokines under the condition of oxygen-glucose deprivation followed by reoxygenation, which is consistent with the *in vivo* effect of endothelial Atg7 knockout.

### Atg7 regulates the expression of pro-inflammatory cytokines through the NF-κB pathway

**Figure 5:**
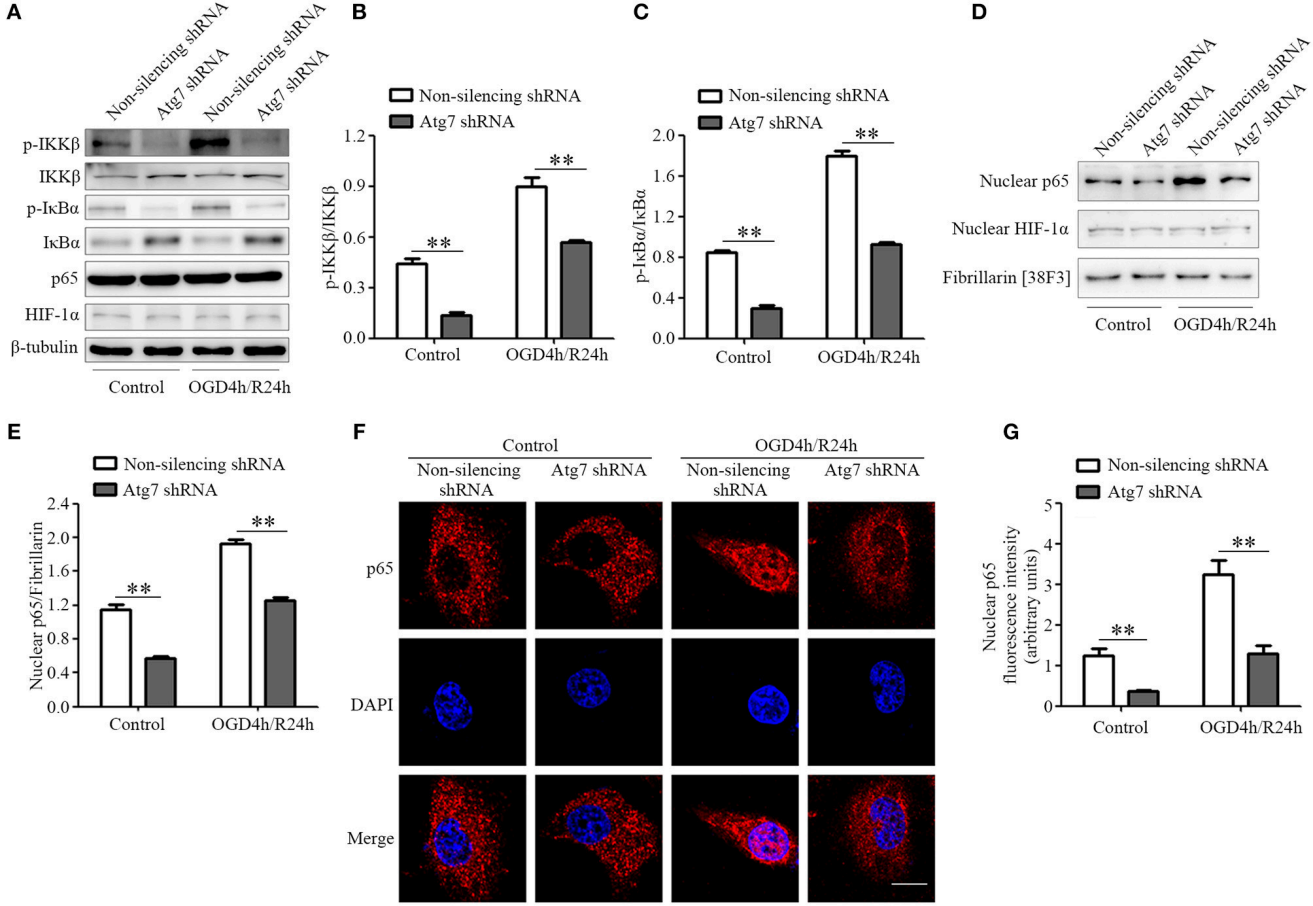
Atg7 regulates the expression of pro-inflammatory cytokines via NF-κB activation. **(A)** The expression of p-IKKβ, IKKβ, p-IκBα, IκBα, p65, and HIF-1α in the Atg7-silenced HBMECs under the normoxia (control) and OGD4h/R24h conditions was determined by western blot analysis, with β-tubulin as the loading control. The representative images were from three independent experiments. **(B,C)** To quantify the results in **(A)**, the band intensities were measured by ImageJ software and the ratio of p-IKKβ/IKKβ **(B)** and p-IκBα/IκBα **(C)** were calculated. ^**^P < 0.01. **(D)** The nuclear extracts were obtained in HBMECs stably transfected with Atg7 shRNA under normoxia and OGD4h/R24h. Then the levels of the NF-κB p65 and HIF-1α were analyzed by western blot. Fibrillarin was detected as the marker protein for nucleus. The representative images were from three independent experiments. **(E)** To quantify the results in **(D)**, the band intensities of p65 in the nuclear fractions were measured with ImageJ software, while the ratio of the nuclear p65 to Fibrillarin was calculated. ^**^P < 0.01. **(F)** Atg7-silenced HBMECs were cultured under either the normoxia or OGD4h/R24h conditions and they were subsequently subjected to immunofluorescence with the primary antibody against p65 (red). DAPI (blue) was used for counterstaining. Scale, 10 μm. **(G)** To quantify the results in **(F)**, the fluorescence intensity of nuclear p65 was measured with ImageJ software. ^**^P < 0.01.

Considering that previous studies revealed the NF-κB to be one of the key molecules regulating the transcription of pro-inflammatory factors (49), we tested whether NF-κB was involved in the Atg7-regulated alteration of pro-inflammatory cytokines. It was demonstrated that IKK (IκB kinase) complex activation promotes IκBα (NF-κB inhibitor) degradation, leading to the nuclear translocation of the NF-κB transcription factor and inducing the expression of inflammatory mediators (50, 51). Therefore, IKK phosphorylation was measured by western blot analysis and we found the IKKβ phosphorylation, the major IKK catalytic subunit for NF-κB activation in response to pro-inflammatory stimuli (50), was reduced upon the Atg7 knockdown (Figures 5A,B). Further results reported the IκBα phosphorylation was significantly decreased by Atg7 knockdown (Figures 5A,C). Moreover, the nuclear translocation of p65, a subunit of the NF-κB transcription complex, was analyzed to evaluate the activation of NF-κB. The nuclear fractions were extracted and the expression of p65 was measured by western blot analysis (Figures 5D,E) and the results described a reduced nuclear distribution of p65 with Atg7 knockdown (Figures 5D,E). Consistently, immunofluorescence revealed that Atg7 knockdown significantly decreased the nuclear localization of p65 (Figures 5F,G).

Previous studies suggested the transcription factor HIF-1 to be a candidate regulator of the transcription of inflammatory factors (52–54). In the current study, both the expression and nuclear distribution of HIF-1α, a catalytic subunit of the HIF-1 heterodimer, were not affected by Atg7 silencing (Figures 5A,D). Additionally, immunofluorescence showed that its nuclear localization was not changed in Atg7-silenced HBMECs, when compared to that in the non-silencing controls (Supplementary Figures 5A,B). These results indicated that NF-κB signaling, but not HIF-1, was specifically exploited by Atg7 to regulate the expression of pro-inflammatory cytokines.

**Figure 6:**
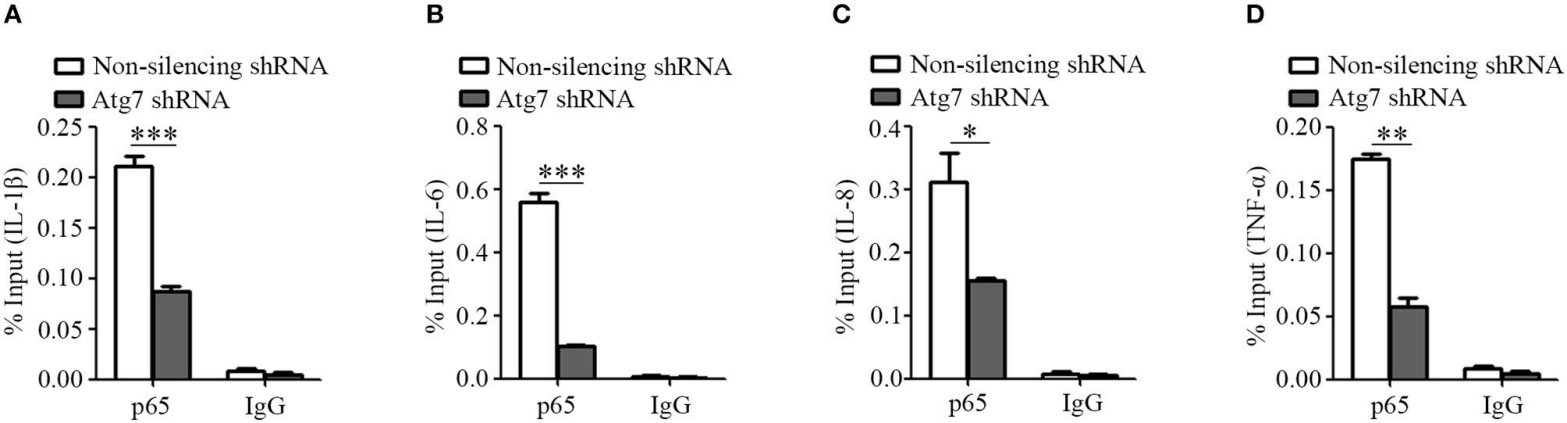
Atg7 silencing reduces the NF-κB binding to the promoter region of inflammatory cytokines. **(A–D)** Atg7-silenced HBMECs were subjected to the ChIP assay using the NF-κB p65 antibody, while the isotype IgG served as the control. The immunoprecipitated DNA fragments were amplified by real-time PCR using the primers flanking the promoter regions of IL-1β, IL-6, IL-8, and TNF-α genes, while the non-silencing shRNA as the control. The expression levels were quantified and the statistical analyses were performed. ^*^P < 0.05, ^**^P < 0.01, ^***^P < 0.001.

Successively, we used a chromatin immunoprecipitation assay to analyze the recruitment of NF-κB to the promoter regions of IL-1β, IL-6, IL-8, and TNF-α genes. We observed that the binding of p65 subunit of NF-κB to the promoter regions of IL-1β, IL-6, IL-8, and TNF-α genes was reduced upon Atg7 knockdown, when compared to the non-silencing controls (Figures 6A–D). These results demonstrated that Atg7 can regulate the mRNA transcription of IL-1β, IL-6, IL-8, and TNF-α via a NF-κB-dependent pathway in brain endothelial cells.

### Atg7 regulates the expression of pro-inflammatory cytokines independent of autophagy

**Figure 7:**
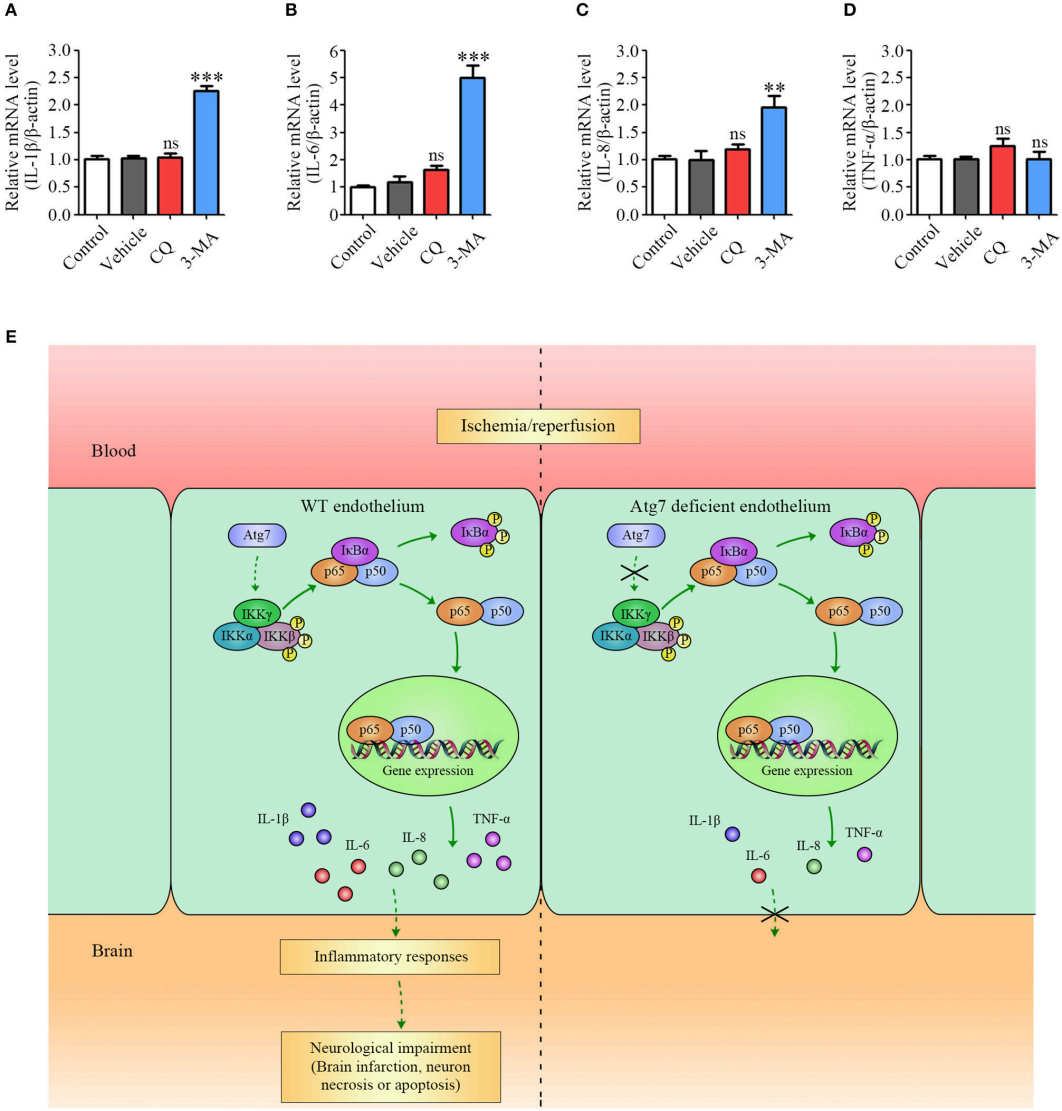
Pro-inflammatory cytokines expression is modulated by Atg7 independent of its autophagic function. **(A–D)** The mRNA levels of IL-1β, IL-6, IL-8, and TNF-α in the HBMECs under the OGD4h/R24h condition were determined by real-time PCR. Where appropriate, cells were further incubated with the following autophagy inhibitors: CQ (25 μM) and 3-MA (5 mM). ^**^P < 0.01, ^***^P < 0.001, ns, no statistical significance. **(E)** Summary model of the proposed mechanism underlying Atg7-regulated ischemic stroke in response to I/R.

Given that Atg7 knockout is known to inhibit autophagy (55, 56), we tested whether the inhibiting effect of Atg7 depletion on pro-inflammatory cytokines expression was caused by Atg7 itself or by the suppressed autophagy. HBMECs were treated with autophagy inhibitors chloroquine (CQ) and 3-methyladenine (3-MA) and the mRNA expression of IL-1β, IL-6, IL-8, and TNF-α was assessed. When HBMECs were treated with CQ, an autophagy pathway inhibitor causing the accumulation of ineffective autophagosomes (57), the mRNA levels of IL-1β, IL-6, IL-8, and TNF-α were not changed under the OGD4h/R24h condition (red column, Figures 7A–D). Following the treatment with 3-MA, a specific inhibitor of both autophagic nucleation and the sequestration phase (58), the mRNA level of TNF-α was not affected by the OGD4h/R24h condition, whereas the mRNA levels of IL-1β, IL-6, and IL-8 were increased (although not decreased) when compared to the vehicle controls (blue column, Figures 7A–D). These data indicated that the autophagic inhibitors, 3-MA and CQ, are not able to recapitulate the reduction in pro-inflammatory cytokines induced by Atg7 depletion in endothelial cells. Overall, these results demonstrated that the inhibiting effect of Atg7 depletion on pro-inflammatory cytokines expression was achieved through the NF-κB-dependent transcriptional regulation, but not via the suppressed autophagy pathway.

## Discussion

Although cerebral blood vessels are one of the first tissues to be affected in the acute phase of an ischemic/reperfusion insult (1), the role of cerebral vessels in the ischemia/reperfusion-induced brain injury remains obscure (19, 59). In the current study, we utilized endothelial-specific Atg7 knockout mice to investigate the role of endothelial Atg7 in the brain lesions occurring in response to ischemia/reperfusion. Both the infarct volume and neuronal death were found to be significantly reduced in those mice with the endothelial Atg7 deletion at 24 h post-ischemia. Additionally, the inflammatory responses induced by I/R, including the expression of pro-inflammatory cytokines (IL-1β, IL-6, IL-8, and TNF-α) and the infiltration of blood-derived neutrophils, were revealed to be effectively alleviated by endothelial Atg7 deletion. Furthermore, although *in vitro* results consistently demonstrated that Atg7 could regulate the transcriptional expression of pro-inflammatory cytokines through NF-κB in brain endothelial cells, the role of Atg7 deficiency in neurons was still controversial (60). Overall, our findings established the protective effect of Atg7 downregulation in cerebral vessels during the inflammatory responses induced by 24-h reperfusion.

The genetic manipulations in blood vessels were previously reported to have a protective effect against the brain I/R injury (20, 61). For example, endothelium-targeted heat shock protein 27 overexpression in mice provides protection against I/R-induced neurological deficits (20). Moreover, endothelial α5 integrin knockout reduced the infarct volumes after stroke in mice (61). In these studies, the protective effects of the genetic manipulations in the cerebral blood vessels were achieved through the preservation of the blood-brain barrier (BBB) integrity (20, 61). However, contrary to these studies, we found that Atg7 eKO alleviated the inflammatory responses induced by I/R injury without affecting the brain microvascular angiogenesis, suggesting that Atg7 depletion exploits alternative pathways in the cerebral vessels to protect against I/R injury. These findings indicated that multiple mechanisms, including the regulation of inflammatory responses and the maintenance of BBB integrity, contribute to the role of cerebral vessels during I/R injury.

Our *in vivo* and *in vitro* results demonstrated that the increased mRNA levels of IL-1β, IL-6, IL-8, and TNF-α in brain endothelial cells upon ischemia/reperfusion were significantly reduced by Atg7 depletion. Furthermore, the results from the ChIP assays revealed that Atg7 silencing attenuated the recruitment of the NF-κB p65 subunit to the promoter of the genes encoding pro-inflammatory cytokines. Our findings therefore unveiled the novel function of Atg7 in the transcriptional regulation of pro-inflammatory cytokines. Interestingly, the role of Atg7 acting as a transcriptional regulator is independent of its activity in autophagy, as the autophagy inhibitors were not able to recapitulate the effect of Atg7 depletion on pro-inflammatory cytokines expression (Figures 7A–D). Regarding the mechanism by which Atg7 modulates NF-κB activation, we reported Atg7 knockdown to reduce the phosphorylation of both IKKβ and IκBα. Given that IKKβ is established to act as the upstream molecule of IκBα to promote NF-κB activation (62, 63), we concluded that Atg7 depletion reduced IKKβ phosphorylation to inhibit NF-κB activation during the inflammatory responses in brain endothelial cells. Given that the protein similarity analysis showed that Atg7 did not contain any kinase domain, it was deduced that the IKKβ phosphorylation was indirectly regulated by Atg7. Several protein kinases have been reported to phosphorylate IKK, including the protein kinase C isoforms, the mitogen-activated protein kinase kinase kinase family members, NIK, AKT, MEKK, COT/TPL-2, and TAK1 (64). Further determining the Atg7 targeting kinase accounting for the IKKβ phosphorylation is of great interest.

Using cultured HBMECs with Atg7 knockdown, we found that Atg7 can regulate the expression of IL-1β, IL-6, IL-8, and TNF-α. Additional results showed this regulation occurred at the transcriptional level via the transcription factor NF-κB. Given the differences between *in vitro* cultured brain endothelial cells and *in vivo* brain microvessels, confirming the results from the Atg7-silenced HBMECs with isolated brain microvessels from Atg7 eKO mice may be appealing in future studies.

Previous studies revealed two putative regulatory mechanisms underlying the inflammatory reactions to I/R injury. Specifically, the primary mechanism involves the activation of brain resident cells, i.e., microglia and astrocytes. In fact, the several inflammatory mediators released by microglia and astrocytes could exacerbate the brain damage followed by I/R (65, 66). In contrast, the other mechanism requires the infiltration of peripheral blood-derived immune cells, particularly neutrophils, carrying pro-inflammatory mediators into the brain during I/R (67). However, our findings suggested that cerebral blood vessels play a pivotal role in the inflammatory responses during the pathological process of I/R, raising a distinct mechanism contributing to the inflammatory reaction of stroke. Therefore, clarifying the timeline of these mechanisms during stroke, including cerebral vascular changes, the activation of resident cells and the infiltration of peripheral neutrophils, will be meaningful in the future.

Furthermore, BBB disruption in the ischemic brain was observed as early as 0.5 h after reperfusion, lasting up to 24 h (19). Early administration of brain microvessels targeting agents, such as the re-engineered erythropoietin that is able to penetrate the BBB, could attenuate the brain I/R damage at the acute phase (24 h after reperfusion) (68). This suggested that the early brain microvessels responses are associated with the acute brain injury induced by ischemia/reperfusion. In the present study, we identified the early protective role of endothelial Atg7 deletion on the brain damage at the acute phase of ischemic stroke. However, identifying the long-term recovery after ischemic stroke still requires further experimental investigations. Thus, it will be interesting to clarify the role of Atg7 in the recovery phase of ischemic stroke in future study.

In summary, the current study investigated the effect of endothelial Atg7 deletion on both the brain damage and the inflammatory reactions in response to I/R injury. Atg7 deficiency was found to have a protective role against the brain damage and to alleviate the inflammatory responses induced by I/R. Further, pro-inflammatory cytokines expression was modulated by Atg7 through the NF-κB-dependent transcriptional regulation (Figure 7E). Our findings established the novel regulatory role of Atg7 in cerebral vessels associated with the inflammatory reactions during stroke. Based on this work, the pharmacological modulation of Atg7 could be a future direction for the clinical treatment of ischemic stroke.

## Author contributions

H-JW conducted most of the experiments and drafted the manuscript. J-YW, D-XL, S-FZ, YL, HL, MB, and W-GF performed part of the experiments. LC provided advice during the manuscript preparation. W-DZ and Y-HC designed the experiments and wrote the final manuscript.

### Conflict of interest statement

The authors declare that the research was conducted in the absence of any commercial or financial relationships that could be construed as a potential conflict of interest.
