# Supplementary material for: Endothelial Atg7 Deficiency Ameliorates Acute Cerebral Injury Induced by Ischemia/Reperfusion
Source: Front Neurol. 2018 Nov 28;9:998. doi: 10.3389/fneur.2018.00998 (PMC6280951; doi:10.3389/fneur.2018.00998)
Supplement: Supplementary file 1 [file Data_Sheet_1.docx]

**Supplementary information**

**Endothelial Atg7 deficiency ameliorates acute cerebral injury induced by ischemia/reperfusion**

Hui-Jie Wang^1^, Jia-Yi Wei^1^, Dong-Xin Liu^1^, Shi-Fang Zhuang^1^, Yuan Li^1^, Hui Liu^1^, Meng Ban^1^, Wen-Gang Fang^1^, Liu Cao^1^, Wei-Dong Zhao^1*^ and Yu-Hua Chen^1*^.

^1^Key Laboratory of Cell Biology, Ministry of Public Health, and Key Laboratory of Medical Cell Biology, Ministry of Education, Department of Developmental Cell Biology, China Medical University, 77 Puhe Road, Shenbei New District, Shenyang, Liaoning Province, 110122, P.R. China.

^*^Corresponding authors:

Yu-Hua Chen
yhchen@cmu.edu.cn

Wei-Dong Zhao
wdzhao@cmu.edu.cn

**Short title/running head**: Endothelial Atg7 KO attenuates I/R injury

**
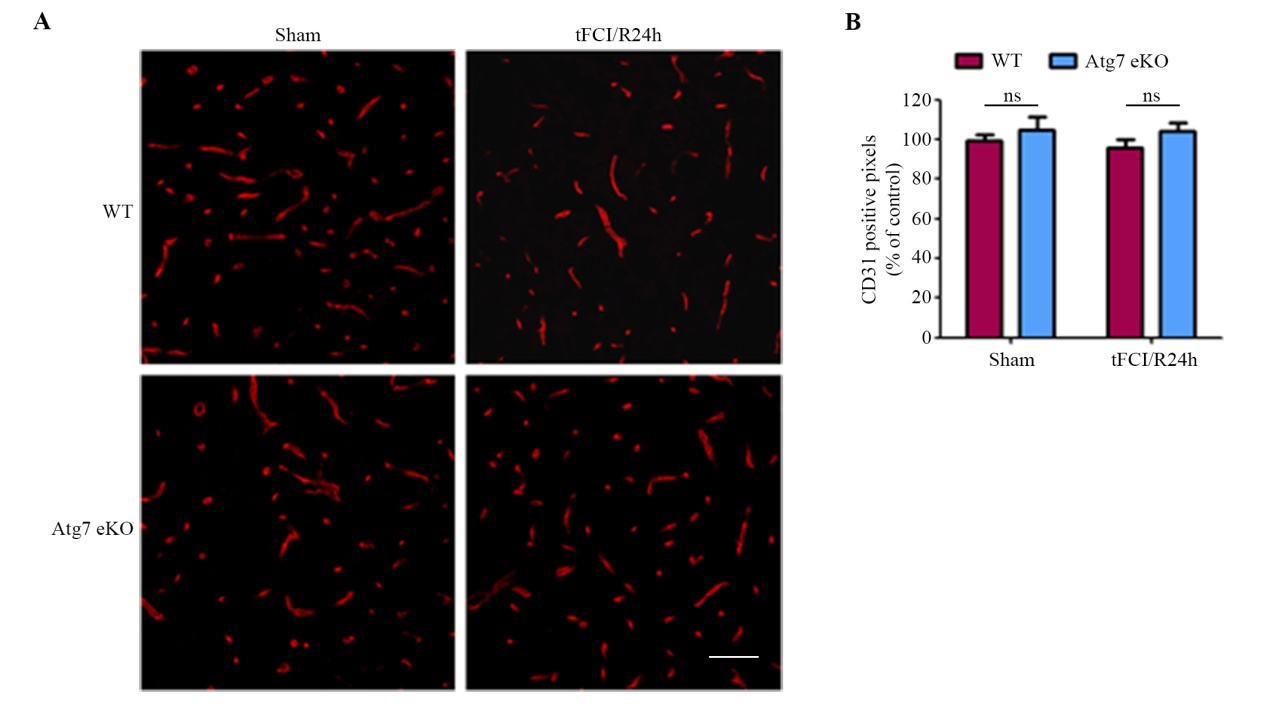
**

**Supplementary Figure 1. Related to Figure 3.** The cerebral vasculature in Atg7 eKO mice after I/R is similar to that in WT mice. (A) Atg7 eKO mice were subjected to either a sham operation or a 1-h ischemia followed by a 24-h reperfusion, while WT littermate mice representing the control. Furthermore, the brain slices were prepared using a cryostat microtome and immunofluorescence was performed with the primary antibody against CD31 (red). Finally, thestained slices were examined under a confocal microscope (n = 10 brain slices from 3 different mice). Scale, 50 µm; (B) To quantify the results in (A), the images were measured by CD31 positive pixels with ImageJ software and the percentage of these pixels was calculated. ns, no statistical significance.


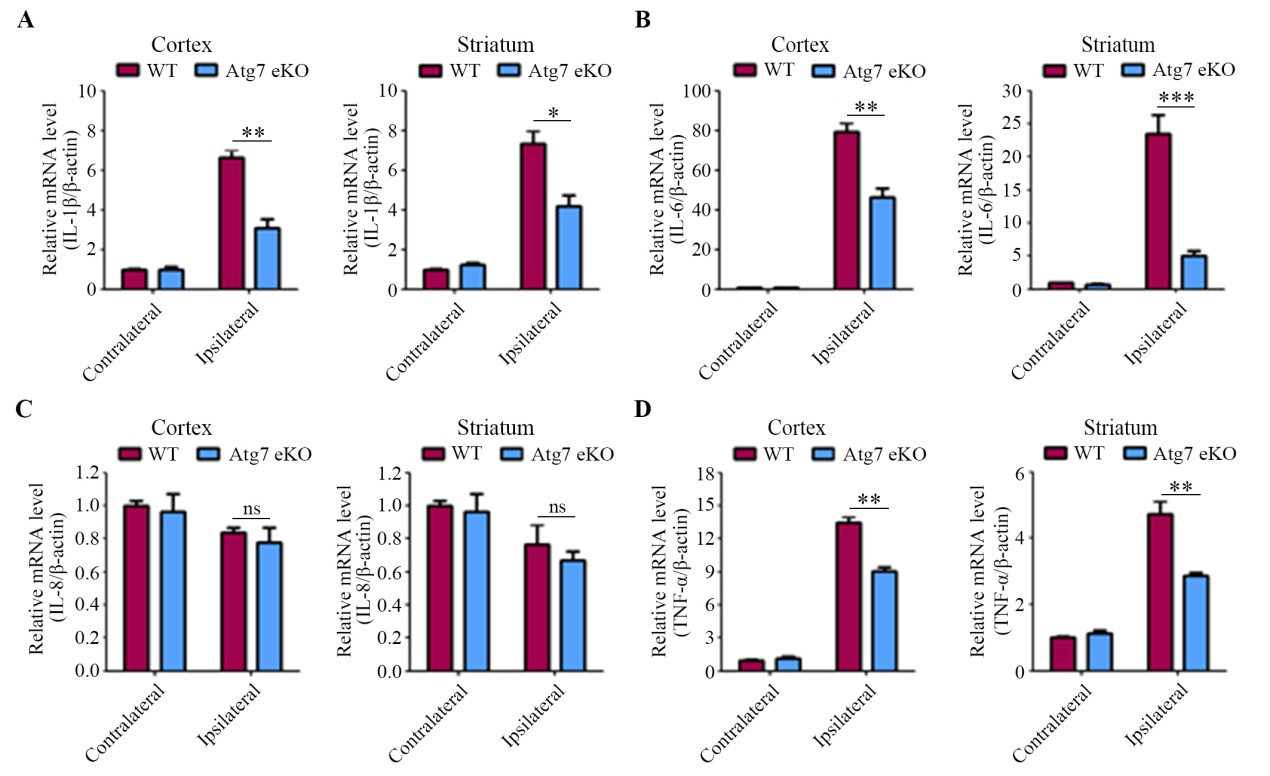


**Supplementary Figure 2.** **Related to Figure 3.** Atg7 eKO attenuates the upregulated expression of pro-inflammatory cytokines in brain induced by I/R. (A ~ D) The mRNA levels of pro-inflammatory cytokines including IL-1β, IL-6, IL-8 and TNF-α in the contralateral and ipsilateral cerebral cortex as well as striatum homogenates from Atg7 eKO mice were detected by real-time PCR, with WT littermate mice used representing the control. **P* < 0.05, ***P* < 0.01, ****P* < 0.001. ns, no statistical significance.


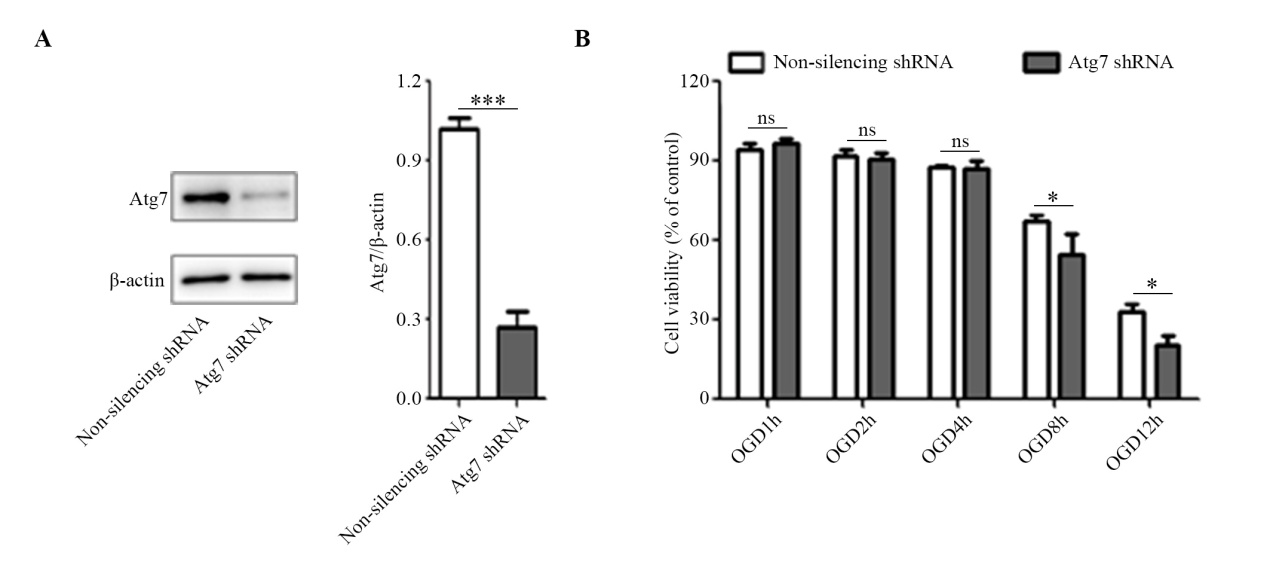


**Supplementary Figure 3. Related to Figure 4.** The effect of Atg7 silencing on the cell viability of HBMECs subjected to oxygen-glucose depletion. (A) HBMECs were stably transfected with Atg7-specific shRNA construct, while HBMECs stably transfected with non-silencing shRNA served as the control. The protein levels of Atg7 were examined by western blot, with β-actin representing the loading control. The relative expression level of Atg7/β-actin was calculated by measuring the band intensity with ImageJ software. ****P* < 0.001; (B) The cell viability of Atg7-silenced HBMECs were was determined using CCK-8 reagent within the 12-h duration of oxygen-glucose depletion (OGD), with non-silencing shRNA as the control. Data from three independent experiments were expressed as the percentage of the control. **P* < 0.05. ns, no statistical significance. Significance was showed at 8 h and 12 h after OGD suggested that Atg7-silenced HBMECs were more sensitive to the OGD lasting longer than 8 h when compared to the control cells.


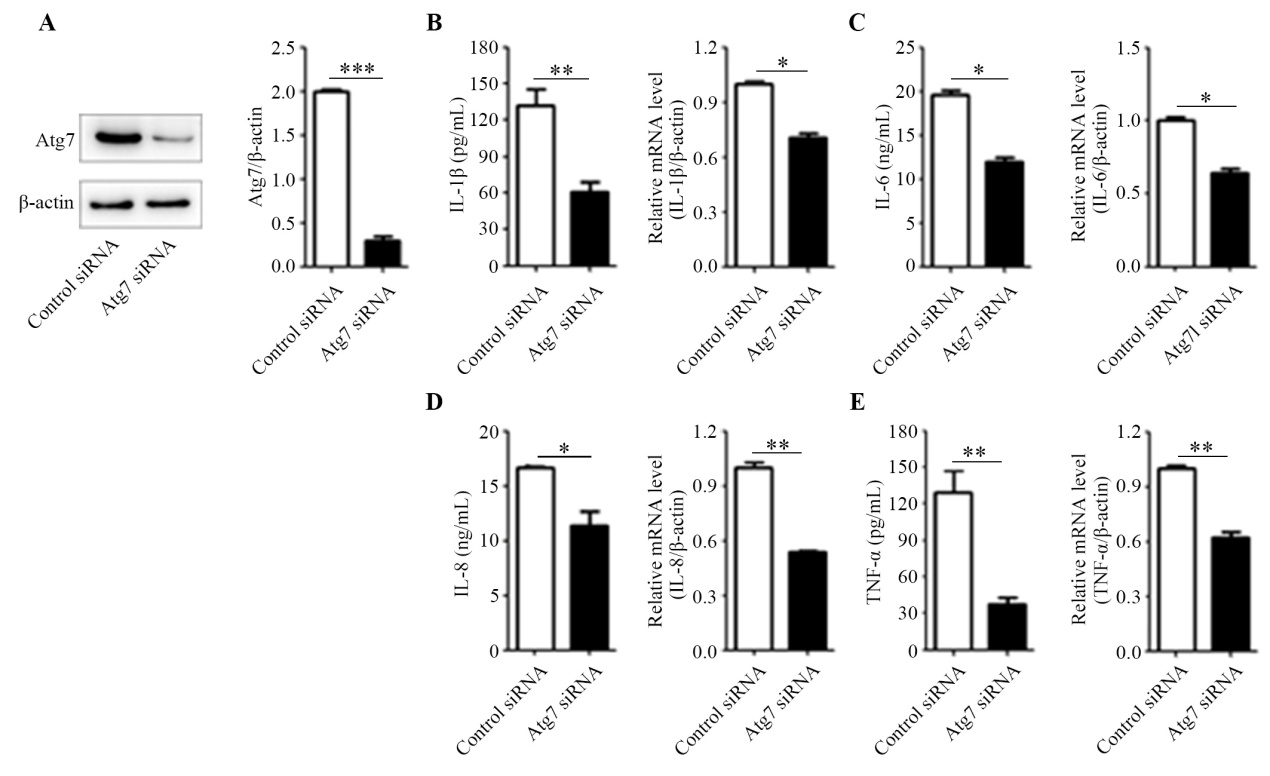


**Supplementary Figure 4.** **Related to Figure 4.** The siRNA-mediated Atg7 knockdown reduces the expression of pro-inflammatory cytokines in brain endothelial cells. (A) HBMECs were transiently transfected with Atg7-specific siRNA, while HBMECs transiently transfected with non-silencing siRNA as the control. The protein levels of Atg7 at 48 h post-transfection were examined by western blot, with β-actin as the loading control. The relative expression level of Atg7/β-actin was calculated by measuring the band intensity with ImageJ software. ****P* < 0.001; (B ~ E) The protein levels of pro-inflammatory cytokines in the HBMECs transfected with Atg7 siRNA were determined through the ELISA, whereas the mRNA levels of pro-inflammatory cytokines were determined by real-time PCR, while HBMECs transiently transfected with non-silencing siRNA as the control. **P* < 0.05, ***P* < 0.01.


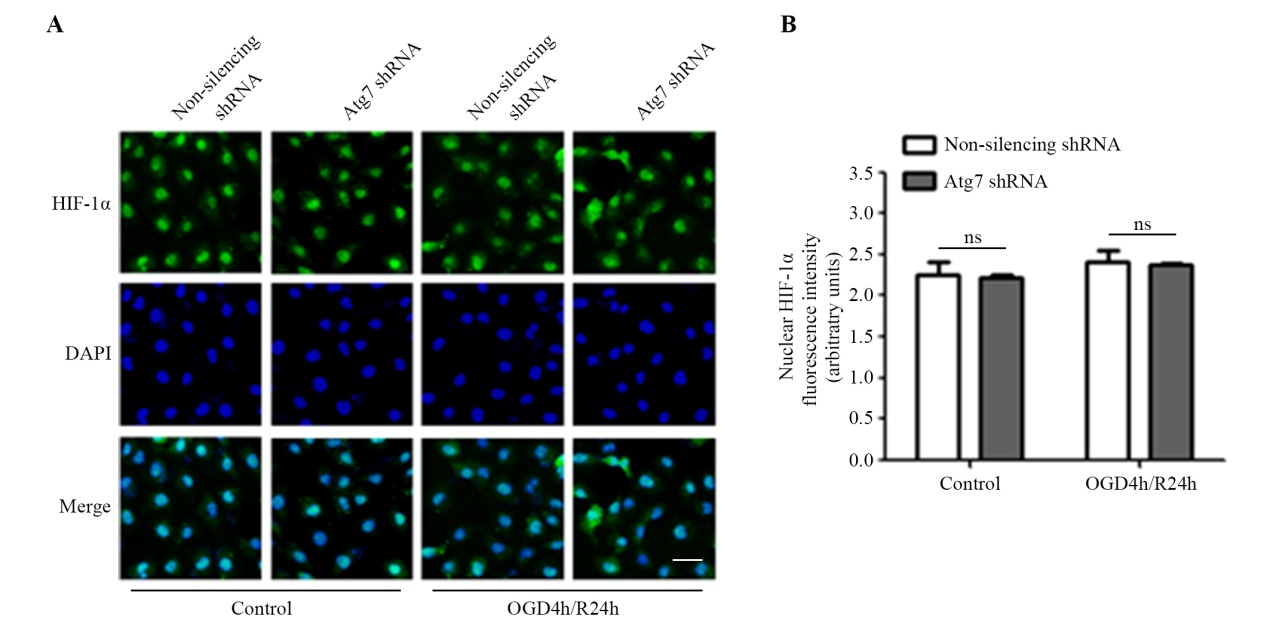


**Supplementary Figure 5. Related to Figure 5.** Effect of Atg7 knockdown on the nuclear distribution of HIF-1α. (A) HBMECs stably transfected with Atg7 shRNA were seeded on cover-slips and immunofluorescence was conducted with the antibody against HIF-1α (green). DAPI (blue) was used for counterstaining. HBMECs stably transfected with non-silencing shRNA were served as the control. Scale bar, 20 µm; (B) To quantify the results in (A), the fluorescence intensity of nuclear HIF-1α was measured with ImageJ software. ns, no statistical significance.

**Supplementary Table 1.** Primers for the pro-inflammatory cytokines detection by real-time PCR

| Target gene | Primer sequence (forward) | Primer sequence (reverse) | Genbank accession no. |
| --- | --- | --- | --- |
| Mouse β-actin | CTGTCCCTGTATGCCTCTG | ATGTCACGCACGATTTCC | NM_007393.5 |
| Mouse IL-1β | AGCATCCAGCTTCAAATC | CATCCCATGAGTCACAGAG | NM_008361.4 |
| Mouse IL-6 | GTTGTGCAATGGCAATTCTGA | GACTCTGGCTTTGTGTCTTTCTTGT | NM_031168.2 |
| Mouse IL-8 | GGTGAAGGCTACTGTTGG | CTGGAGTCCCGTAGAAAA | NM_011339.2 |
| Mouse TNF-α | CTCAGCCTCTTCTCATTCCT | TCCTCCACTTGGTGGTTT | NM_013693.3 |
| Human β-actin | CACCAACTGGGACGACAT | ACAGCCTGGATAGCAACG | NM_001101.4 |
| Human IL-1β | CCAGTGAAATGATGGCTTAT | TGTAGTGGTGGTCGGAGA | NM_000576.2 |
| Human IL-6 | ACACAGACAGCCACTCACCTC | AGCATCCATCTTTTTCAGCCA | NM_000600.4 |
| Human IL-8 | ACTCCAAACCTTTCCACC | CTTCTCCACAACCCTCTG | NM_000584.3 |
| Human TNF-α | ACACCATCAGCCGCATCG | AGTCGGTCACCCTTCTCC | NM_000594.3 |

**Supplementary Table 2.** Primers used in the ChIP-qPCR analysis

| Target promoter gene | Primer sequence (forward) | Primer sequence (reverse) | Genbank accession no. |
| --- | --- | --- | --- |
| Human IL-1β promoter | TACAGACAGGGAGGGCTATT | GTGGGACAAAGTGGAAGACA | X04500.1 |
| Human IL-6 promoter | ACCCTCACCCTCCAACAAAG | GCCTCAGACATCTCCAGTCC | AF048692.1 |
| Human IL-8 promoter | GGGCCATCAGTTGCAAATC | GCTTGTGTGCTCTGCTGTCTC | M28130.1 |
| Human TNF-α promoter | ATATGGCCACACACTGGGGC | GGGCTTGGTGGCAGGCTTGA | AY274889.1 |
